# miRNA‐130b‐5p promotes hepatic stellate cell activation and the development of liver fibrosis by suppressing SIRT4 expression

**DOI:** 10.1111/jcmm.16766

**Published:** 2021-07-17

**Authors:** Hao Wang, Zeng Wang, Yirui Wang, Xiangcheng Li, Wenjie Yang, Song Wei, Chengyu Shi, Jiannan Qiu, Ming Ni, Jianhua Rao, Feng Cheng

**Affiliations:** ^1^ Hepatobiliary/Liver Transplantation Center The First Affiliated Hospital with Nanjing Medical University Nanjing China; ^2^ Key Laboratory of Liver Transplantation Chinese Academy of Medical Sciences Nanjing China; ^3^ Key Laboratory of Living Donor Liver Transplantation National Health Commission (NHC) Nanjing China; ^4^ School of Medical Southeast University Nanjing China

**Keywords:** hepatic fibrosis, HSC activation, miR‐130b‐5p, SIRT4

## Abstract

Liver fibrosis is a progressive disease accompanied by the deposition of extracellular matrix (ECM). Numerous reports have demonstrated that alterations in the expression of microRNAs (miRNAs) are related to liver disease. However, the effect of individual miRNAs on liver fibrosis has not been studied. Hepatic stellate cells (HSCs), being responsible for producing ECM, exert an important influence on liver fibrosis. Then, microarray analysis of non‐activated and activated HSCs induced by transforming growth factor β1 (TGF‐β1) showed that miR‐130b‐5p expression was strongly up‐regulated during HSC activation. Moreover, the progression of liver fibrosis had a close connection with the expression of miR‐130b‐5p in different liver fibrosis mouse models. Then, we identified that there were specific binding sites between miR‐130b‐5p and the 3′ UTR of Sirtuin 4 (SIRT4) via a luciferase reporter assay. Knockdown of miR‐130b‐5p increased SIRT4 expression and ameliorated liver fibrosis in mice transfected with antagomiR‐130b‐5p oligos. In general, our results suggested that miR‐130b‐5p promoted HSC activation by targeting SIRT4, which participates in the AMPK/TGF‐β/Smad2/3 signalling pathway. Hence, regulating miR‐130b‐5p maybe serve as a crucial therapeutic treatment for hepatic fibrosis.

## 1 INTRODUCTION

Increasing hepatic cirrhosis deaths is an intractable problem in the world.^1^ As a precursor to cirrhosis, liver fibrosis has been extensively studied. The denominator of liver fibrosis is excess deposition of extracellular matrix (ECM) and disorder of the lobular structure of the liver, and the cause of hepatic fibrosis is exposed to long‐term chronic liver injury, such as hepatitis virus infection, alcohol abuse–induced liver injury or non‐alcoholic steatohepatitis.^2^, ^3^ Hepatic stellate cells (HSCs), which produce a mass of ECM after being activated, act an essential role during the progression of liver fibrosis.^4^ In the presence of external stimuli and liver injury, these cells go from quiescent to active. In activated HSCs, fibrotic marker gene expression is increased.^5^, ^6^ Currently, the mechanism of HSC activation is unclear and needs to be explored.

MicroRNAs (miRNAs), a subclass of short, non‐coding RNAs, could regulate target mRNA degradation and protein translation.^7^ In recent years, numerous reports have indicated that miRNAs are important regulators of gene expression in various liver diseases.^8^ For example, exosomal miR‐21 contributes to tumour progression in hepatocellular carcinoma.^9^ Alcohol regulates miR‐148a expression to induce hepatocyte pyroptosis via FoxO1.^10^ miR‐192‐5p increases the degree of liver damage.^11^

Then, how miRNAs involved in HSC activation and the relationship between miRNAs and HSCs have been reported in several studies.^12^, ^13^ Therefore, miRNAs play a key role in HSC activation.

The aim of this study was to explore the effect of miR‐130b‐5p on liver fibrosis. First, the data suggested that miR‐130b‐5p expression was significantly up‐regulated during the process of HSC activation using microarray analysis. Next, we detected the expression of miR‐130b‐5p in experimental hepatic fibrosis models and in clinical patient samples. Consistently, miR‐130b‐5p expression was increased. Then, we found that SIRT4 may be the target of miR‐130b‐5p according the predicting software. Recent study had been demonstrated that SIRT4 could alleviate the HFD‐induced liver fibrosis. EX‐527, a SIRT1 inhibitor, could inhibit the progression of high‐fat diet (HFD)–induced hepatic fibrosis via up‐regulating SIRT4.^14^ In this study, we identified a novel regulatory mechanism of miR‐130b‐5p–mediated SIRT4/AMPK inactivation on the HSC activation in liver fibrosis diseases. Further experiments revealed that miR‐130b‐5p promoted liver fibrosis, mechanistically activating HSCs and promoting its proliferation by repressing SIRT4 expression. In addition, in vivo administration of antagomiR‐130b‐5p relieved hepatic fibrosis. All in all, these results identify that the miR‐130b‐5p/SIRT4 axis as a possible novel regulator of HSC‐mediated liver fibrosis and a potential therapeutic target.

## 2 MATERIALS AND METHODS

### 2.1 Clinical liver sample collection

Liver specimens were collected from patients with fibrosis or other liver diseases (the remote tissues of liver haemangioma) in the First Affiliated Hospital of Nanjing Medical University. All samples were instantly store in liquid nitrogen for rapid freezing after surgical resection. Informed consent was obtained from all subjects. All methods followed the relevant guidelines and regulations of Ethics Committee of Affiliated Hospital of Nanjing Medical University.

### 2.2 Cell line and cell culture

HSC‐T6 cells were obtained from the Cell Center of Shanghai Institutes for Biological Sciences. HSCs were maintained in DMEM (KeyGEN Biotech, China) with 10% foetal bovine serum (FBS: WISENT, Canada) and incubated 5% CO_2_ at 37°C. HSCs were cultured in serum‐free DMEM supplemented with TGF‐β1 (10 ng/mL) for 0, 3, 6, 12 or 24 hours for activation.

### 2.3 Western blotting analysis

Protein was obtained from cells in RIPA lysis buffer (Beyotime Biotechnology) with protease inhibitors and phosphatase inhibitors. Extracted protein was subjected to SDS‐polyacrylamide gel electrophoresis and transferred to polyvinylidene fluoride (PVDF) membranes (Bio‐Rad). After blocking at room temperature for 2 hours with 5% non‐fat dried milk, membranes were incubated with primary antibody overnight at 4°C. Membranes were then incubated with the appropriate secondary antibodies for 2 hours at room temperature. The protein band was measured in an electro‐chemiluminescence detection system (Thermo Fisher Scientific). Primary antibodies used were as follows: SIRT4 (Abcam), α‐SMA, Collagen I, TIMP‐1, TGF‐β, AMPK, p‐AMPK, Smad3, p‐Smad3, Smad2, p‐Smad2 and β‐actin rabbit mAbs (1:1000; Cell Signaling Technology). The secondary antibodies, including HRP‐conjugated goat anti‐rabbit immunoglobulin G (IgG) or goat anti‐mouse IgG (Cell Signaling Technology, MA, United States), were diluted 1:2000. The proteins were detected using ECL chemiluminescence (GE Amersham, Arlington Heights, IL).

### 2.4 RNA extraction and quantitative RT‐PCR

Total RNA was extracted from tissue or cells with TRIzol reagent (Invitrogen). The Reverse Transcription Kit (Vazyme) was performed to reverse RNA and synthesize cDNA. SYBR green (Vazyme) was used to perform mRNA amplification and cDNA quantification. The levels of U6 and miR‐130b‐5p were detected by using TaqMan miRNA assay system (Life Technologies Corporation). Gene expression was normalized to snRNA U6 or β‐actin expression, respectively. Primer sequences are shown in Table S1.

### 2.5 miR‐130b‐5p and SIRT4 transfection in vitro or in vivo

miR‐130b‐5p mimics, scrambled miRNA (SCR‐miRNA), antagomiR control (NC‐miR), antagomiR‐130b‐5p, SIRT4 lentivirus (shSIRT4), SIRT4 lentivirus (LV‐SIRT4) and control lentivirus were purchased from GenePharma. NC‐miR and antagomir‐130b‐5p were transfected into HSC‐T6 cells for 48 hours, and cells were collected for further experiment. HSC‐T6 cells were transfected with lentiviruses using Lipofectamine 2000 for 48 hours (Invitrogen). Mice were treated with NC‐miR and antagomiR‐130b‐5p (Gema) at a concentration of 20 nmol/200 µL via the tail vein injection. The mice were first treated with CCl_4_ or treated by HFD for 2 weeks and then injected with NC‐miR and antagomiR‐130b‐5p twice a week. Mice were killed after the end of treatment. Mice that underwent BDL were treated with antagomiR‐130b‐5p and NC‐miR twice a week via the tail vein injection. Control mice were treated with NC‐miR and antagomiR‐130b‐5p via tail vein twice a week. Mice were killed after the end of treatment. Liver tissues were collected for further experiments.

### 2.6 Microarray analysis

Total RNA was isolated from quiescent (induced with TGF‐β1 for 0 hour) and activated (induced with TGF‐β1 for 24 hours) HSC‐T6 cells, respectively, with TRIzol reagent (Life Technologies). RNA was purified with the RNA‐quick Purification kit (YiShan Biotech). Microarray analysis was performed by Shanghai Biotechnology Corporation. The analysis of scanned images was conducted by using anscaAgilent Feature Extraction software (version 10.7).

### 2.7 Cell cycle assay and apoptosis

Cell cycle experiments were performed using the Cell Cycle Analysis Kit (Beyotime). HSCs were collected and digested with trypsin. Then, after washing with pre‐cooled PBS, the cells were fixed in 70% ethanol at −20°C overnight. After incubating cells with RNase (50 µg/mL) and washing them twice with pre‐cooled PBS, flow cytometry analysis (FCM) was performed. For cell cycle analysis, propidium iodide (PI) staining solution (500 mL) was used to stain cells. The cells were incubated with PI (Sigma) and Annexin V‐FITC (BD Biosciences) to stain apoptotic cells. The right upper quadrant of FACS data and the right lower quadrant of FACS data add up to the total apoptosis index.

### 2.8 Mouse liver fibrosis models

Eight‐week‐old male C57BL/6 mice were obtained from the Animal Center of Nanjing Medical University (NJMU) and were housed under a specific pathogen–free conditions. For the olive oil group and CCl_4_ group, mice received olive oil (2 mL/kg) or CCl_4_ (10% in olive oil, 2 mL/kg) by intraperitoneally injection twice a week for 8 weeks. For the sham group and BDL group, male mice were subjected to sham or BDL surgery. For the chow group (normal diet) and HFD group, healthy mice were fed a high‐fat diet (protein, 18.1%; fat, 61.6%; carbohydrates, 20.3%; D12492; Research Diets) continuously for 24 weeks. Mice administered a normal control (NC) diet (protein, 18.3%; fat, 10.2%; carbohydrates, 71.5%; D12450B; Research Diets) served as controls.

### 2.9 Liver histopathology and fibrosis measurement

Formaldehyde‐fixed, paraffin‐embedded liver sections were stained with Masson and Sirius red to evaluate the extent of liver fibrosis. Dehydrated slides were scanned and imaged. The staining was further quantified by a pathologist. The positive staining area of Masson, Sirius red and α‐SMA immunohistochemistry staining was measured using ImageJ software and quantified in a bar graph. The percentages of the Masson‐positive, Sirius red–positive and α‐SMA‐positive areas were calculated from five fields for each liver slice.

### 2.10 Immunohistochemistry

Formalin‐fixed and paraffin‐embedded liver samples were deparaffinized, rehydrated and subjected to antigen retrieval. Samples were incubated with a primary antibody against α‐SMA (1:500; Cell Signaling Technology) or SIRT4 (1:500; Abcam) overnight at 4°C. Then, the samples were washed with pre‐cooled PBS and incubated with HRP‐polymer‐conjugated secondary antibody at 37°C for 1 hour. 3,3′‐Diaminobenzidine tetrahydrochloride staining was performed to evaluate the expression of α‐SMA. The nuclei were counterstained with haematoxylin.

### 2.11 Immunofluorescence (IF) staining

HSC‐T6 cells were incubated with anti‐α‐SMA primary antibody (Cell Signaling Technology), and then, the cells were treated with secondary goat anti‐rabbit Texas Red–conjugated IgG (Sigma) and DAPI. After washing the slides twice with pre‐cooled PBS, we used confocal microscopy (ZEISS, Oberkochen) to obtain fluorescence images of the cells according to the manufacturer's instructions.

### 2.12 Cell viability and colony formation assay

Hepatic stellate cells were plated in a 96‐well plate (2000 cells /well) and treated with DMEM (10% FBS) for 5 days. Each well was treated with 10 μL Cell Counting Kit‐8 (CCK‐8) solution (Dojindo) and incubated for 2 hours at 37°C, and cell viability was assessed by measurement of the optical density measured at 450 nm. For the colony formation assay, untreated and treated HSCs were seeded in 6‐well plates (1000 cells/well) and maintained in DMEM (10% FBS) for 2 weeks. The colonies were fixed with 70% ethanol and stained with 0.1% crystal violet. Proliferating colonies (>50 cells/colony) were counted.

### 2.13 Luciferase reporter assay

HSC‐T6 cells were co‐transfected with 0.12 µg of pGL3‐SIRT4 3′‐UTR reporter plasmid (Ambion) containing the wild‐type or mutated miR‐130b‐5p binding sequence together with 40 nM of miR‐130b‐5p mimics or negative control oligoribonucleotides using Lipofectamine 3000 (Invitrogen). Then, luciferase activities were detected using the Dual‐luciferase Reporter Assay System (Promega). A Renilla luciferase expression plasmid was transfected into HSC‐T6 cells as a negative control.

### 2.14 Statistical analysis

Statistical analyses were performed using GraphPad Prism software or Statistical Package for the Social Sciences (SPSS) software version 19.0 and displayed as means ± standard deviation (SD). Differences between two groups were analysed using Student's *t* test. For multiple comparisons, the results were corrected by the Bonferroni method. *P* < .05 was considered statistically significant (*P < .05, **P < .01, ***P < .001).

## 3 RESULTS

### 3.1 miR‐130b‐5p is up‐regulated during HSCs activation

**FIGURE 1:**
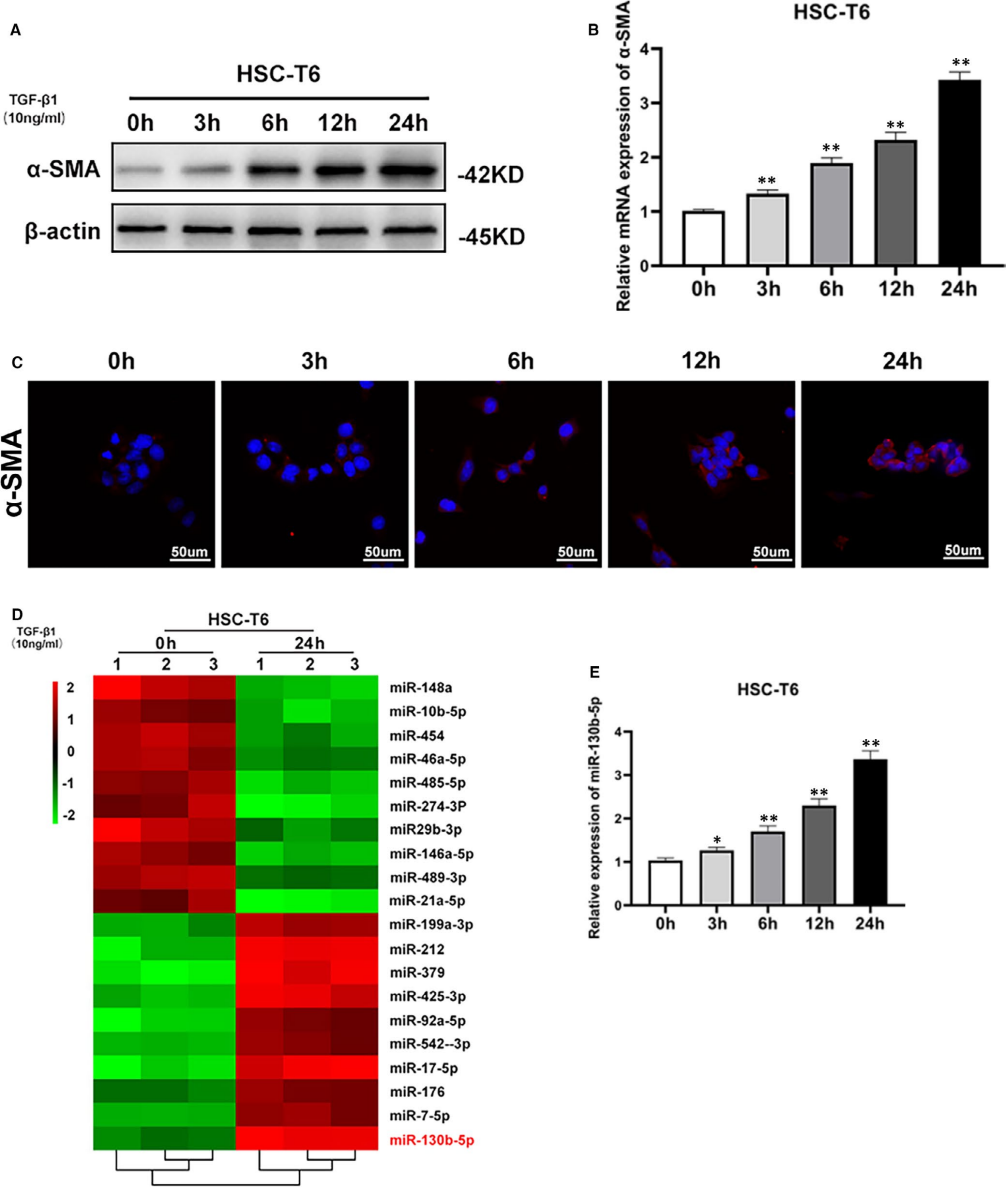
Up‐regulated expression of miR‐130b‐5p during HSC activation. A, The protein expression level of α‐SMA was increased in activated HSC‐T6 cells induced by 10 ng/mL TGF‐β1 at different time points. B, The mRNA expression of α‐SMA was increased in activated HSC‐T6 cells induced by 10 ng/mL TGF‐β1 at different time points. C, α‐SMA (red) was identified by immunofluorescence assays in HSC‐T6 cells induced by 10 ng/mL TGF‐β1 at different time points. D, MicroRNA microarray analysis results in quiescent and activated HSCs. E, The expression level of miR‐130b‐5p was detected by quantitative real‐time PCR. F, The expression level of miR‐130b‐5p in activated HSCs was detected at different time points. Data represent means ± SEM of at least three independent experiments. *P < .05, **P < .01 and ***<.001

First, we cultured activated HSCs under the induction of TGF‐β and detected the expression levels of α‐SMA. TGF‐β1‐induced activation of HSC‐T6 cells was connected with α‐SMA expression up‐regulation (Figure 1A‐C). To identify microRNAs that may regulate HSC activation, total RNA extracted from HSCs activated by 10 ng/mL TGF‐β1 for 0 or 24 hours was performed. Ten up‐regulated and 10 down‐regulated miRNA species after HSC activation are displayed in Figure 1D. We detected the expression level of several miRNAs (> 2‐fold changes) by qPCR (Figure S2). Among these differentially expressed miRNAs, miRNA‐130b‐5p was most significantly up‐regulated miRNAs in HSCs. The expression of this miRNA was examined by RT‐qPCR analysis, which indicated a time‐dependent up‐regulation under the stimulus of TGF‐β1 in activated HSCs (Figure 1E). These findings indicated that miRNA‐130b‐5p expression is up‐regulated during HSC‐T6 cell activation.

### 3.2 Up‐regulated miRNA‐130b‐5p expression in multiple liver injury models

**FIGURE 2:**
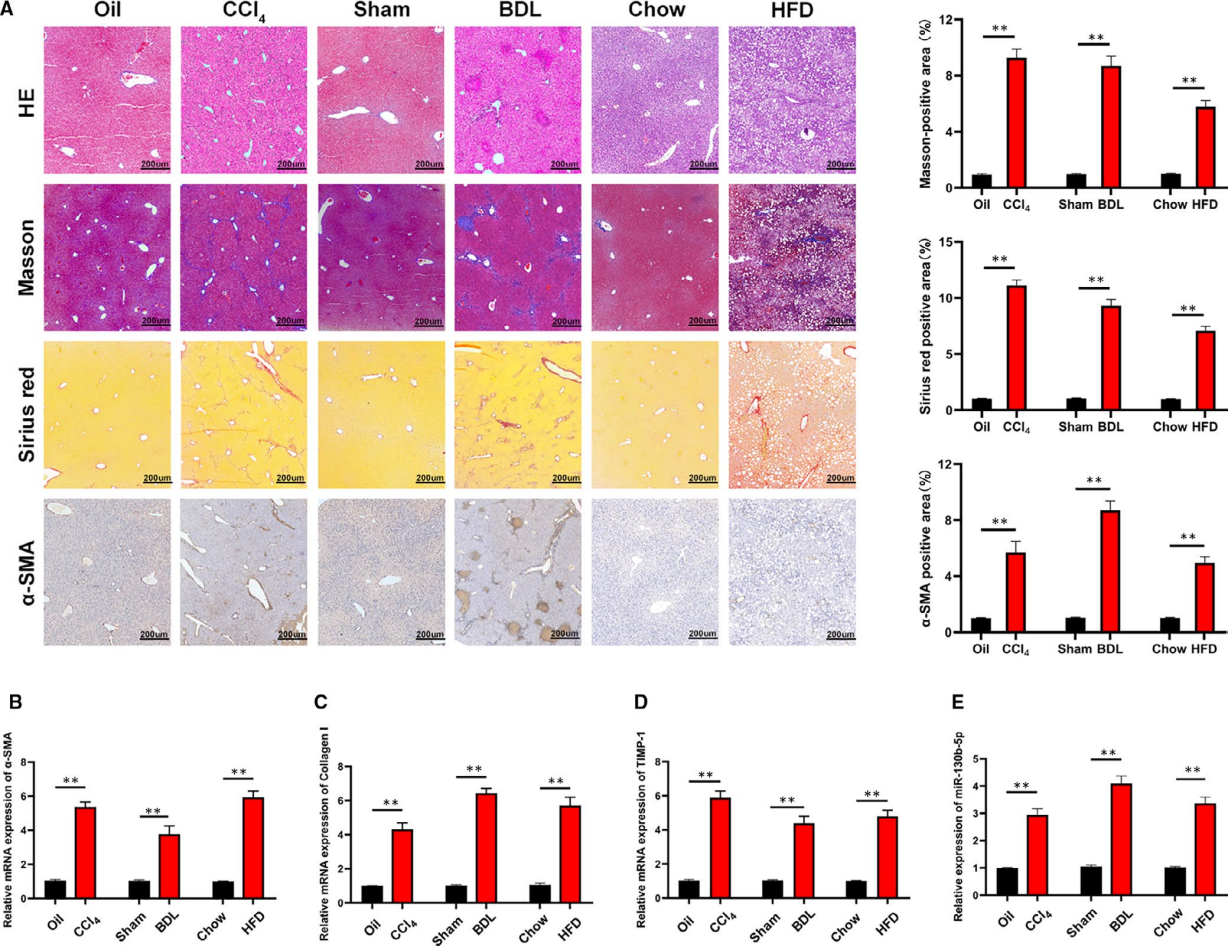
miR‐130b‐5p is up‐regulated in multiple liver fibrosis models. A, Liver sections stained with H&E and Masson staining for assessing liver fibrosis in mice treated with olive oil or CCl_4_ for 8 wk, sham or BDL for 2 wk and chow or HFD for 24 wk (n = 6/group; original magnification ×100; scale bars, 200 μm). The quantification of Masson‐positive, α‐SMA‐positive and Sirius red‐positive fibrosis areas in the livers from mice. B, The mRNA level of α‐SMA was measured in liver tissue of treated mice by quantitative real‐time. n = 6/group. C, Collagen I expression was measured in liver tissue of treated mice at mRNA level. n = 6/group. D, TIMP‐1 was detected in liver tissue of treated mice at mRNA level. n = 6/group. E, The expression level of miR‐130b‐5p was examined in liver tissue of treated mice by quantitative real‐time PCR. n = 6/group. Graph represents mean ±SEM. *P < .05, **P < .01, and ***P < .001

The mice were exposed to CCl_4_ subcutaneous injection and subjected to BDL surgery. Since non‐alcoholic fatty liver disease (NASH) has been one of the important causes that led to hepatic fibrosis, we used HFD to induce liver fibrosis model. The degree of liver fibrosis was also validated by H&E, Masson and α‐SMA staining, which revealed obvious fibrous tissues (Figure 2A). In addition, the RT‐PCR data revealed that the level of a‐SMA, Collagen I and TIMP‐1, which are genes associated with fibrosis, were significantly increased in liver tissues of treated mice compared with those of control mice (Figure 2B‐D). Furthermore, miRNA‐130b‐5p expression was strongly up‐regulated in the livers with fibrosis (Figure 2E). A recent study indicated that down‐regulated miR‐130b‐5p expression could reduce hepatic lipid accumulation.^15^ These results support that miRNA‐130b‐5p likely has a significant effect on the development of liver fibrosis.

### 3.3 miR‐130b‐5p regulates the activation, proliferation and apoptosis of HSCs

**FIGURE 3:**
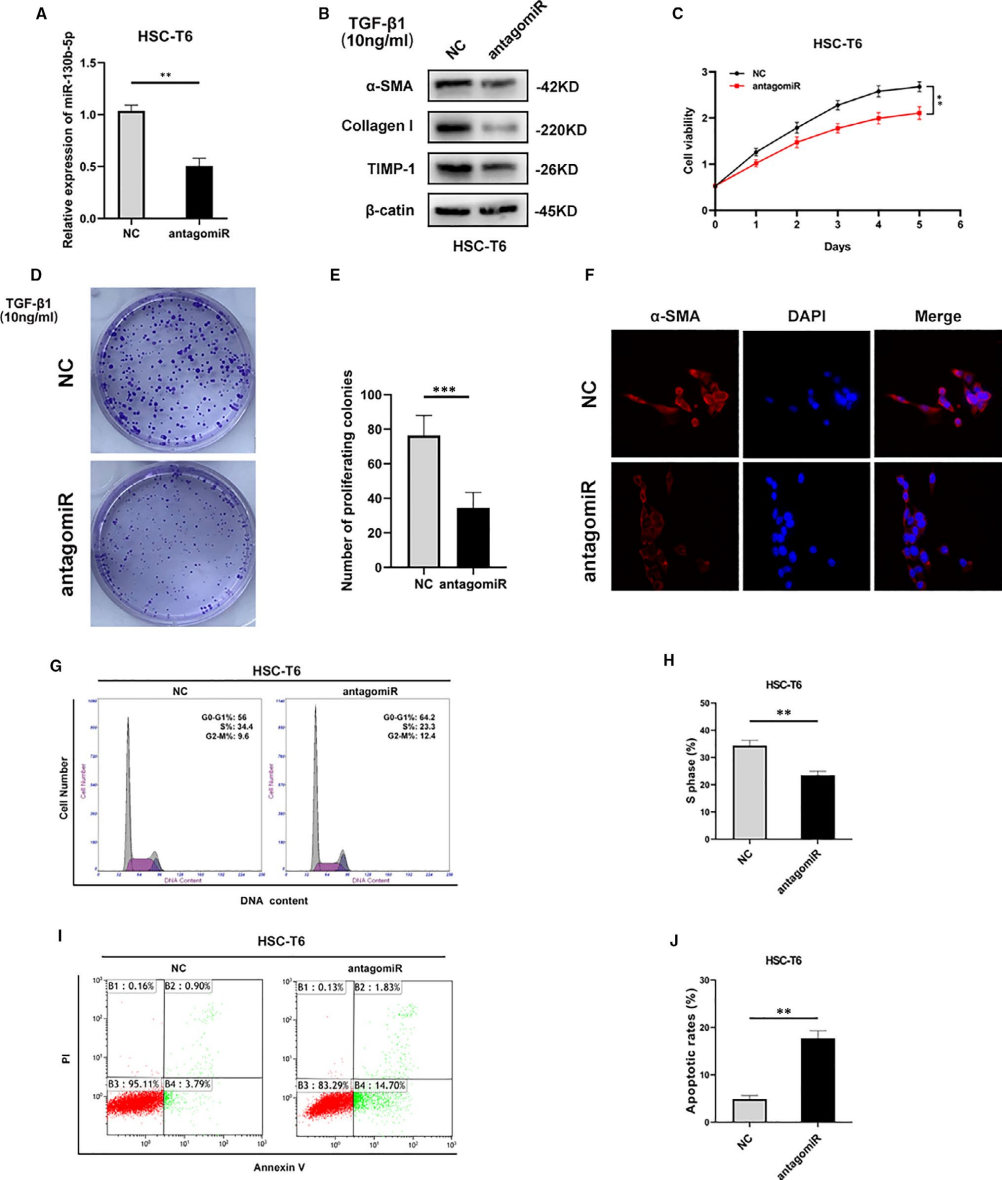
miR‐130b‐5p regulates the activation and proliferation of HSCs. (A) The expression level of miR‐130b‐5p was detected in HSCs after transfection with either antagomir‐130b‐5p or NC‐miR. (B) The protein levels of fibrotic genes, including α‐SMA, Collagen I and TIMP‐1, were detected by Western blotting in HSC‐T6 cells induced by 10 ng/mL TGF‐β for 24 h. (C) Proliferation of HSCs transfected with antagomir‐130b‐5p or NC‐miR was assessed using cell viability assays, (D) colony assays and (E) the quantification. (F) α‐SMA (red) was identified by immunofluorescence assays in HSC‐T6 cells transfected with antagomir‐130b‐5p or NC‐miR under the stimulus of TGF‐β (10 ng/mL). (G) The cell cycle distribution of cells transfected with NC‐miR or antagomir‐130b‐5p measured by flow cytometry and (H) the quantification. (I) The cell apoptosis of cells transfected with NC‐miR or antagomiR‐130b‐5p was measured by flow cytometry and (J) the quantification. Data represent means ± SEM of at least three independent experiments. *P < .05, **P < .01 and ***<.001. *P < .05, **P < .01 and ***P < .001

To evaluate the functional role of miR‐130b‐5p in fibrogenesis, HSCs were transfected with antagomiR‐130b‐5p or NC‐miR (Figure 3A). In order to assess the effect of the transfection treatment on the results of cell proliferation, cell apoptosis and immunofluorescence, the experiment were made and data were presented in Figure S3. Pronounced down‐regulation of the expression of fibrotic marker (including a‐SMA, TIMP‐1 and Collagen I) was detected by RT‐PCR and Western blot in the group with decreased miR‐130b‐5p expression compared with the control group, demonstrating that miRNA‐130b‐5p promoted the activation of HSCs (Figure 3B). In addition, cell growth was suppressed after miR‐130b‐5p expression was suppressed by antagomiR‐130b‐5p in activated HSCs, as determined by the cell viability assay and colony formation assay (Figure 3C‐E). Immunofluorescence analysis suggested down‐regulation of α‐SMA expression in HSC‐T6 cells transfected with NC‐miR or antagomiR‐130b‐5p under the treatment of TGF‐β (10 ng/mL) (Figure 3F). Next, we studied whether miRNA‐130b‐5p regulate the cell cycle of HSC‐T6 cells. Compared to the negative control, antagomiR‐130b‐5p obviously inhibited HSC proliferation and reduced the percentage of HSCs in the S and G2 phases under the treatment of TGF‐β (10 ng/mL) (Figure 3G,H and Figure S1A). Moreover, knockdown of miR‐130‐5p promoted apoptosis of HSCs under the treatment of TGF‐β (10 ng/mL) (Figure 3I,J). These results revealed that miR‐130b‐5p positively regulates activation, proliferation and apoptosis of HSCs.

### 3.4 miR‐130b‐5p suppresses SIRT4 expression

**FIGURE 4:**
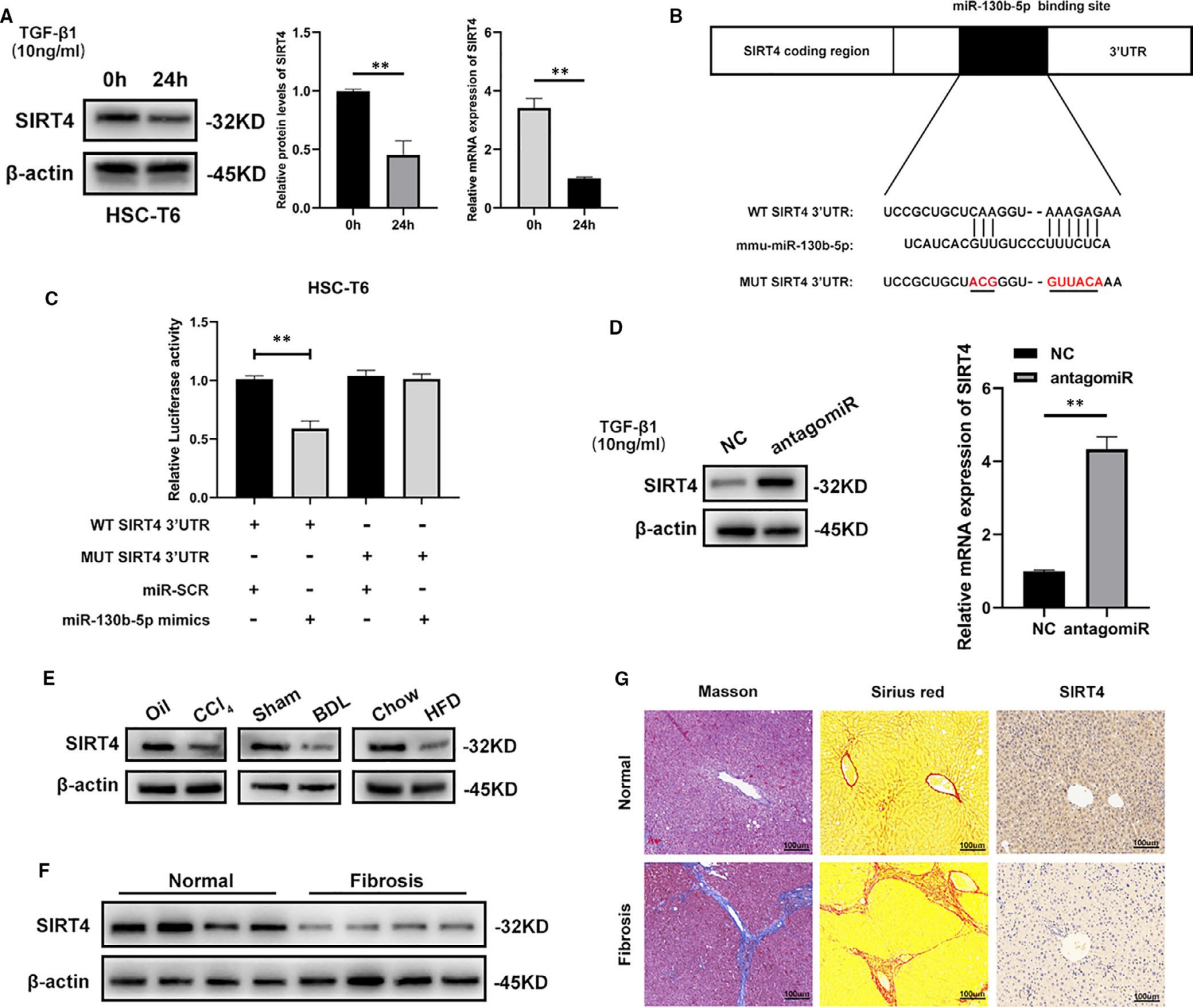
miR‐130b‐5p targets SIRT4 and regulate its expression. A, The protein levels and mRNA expression of SIRT4 in HSC‐T6 cells induced by 10 ng/mL TGF‐β for 0 and 24 h. B, Predicted miR‐130b‐5p targeting sequence in SIRT4 3′UTR (WT SIRT4 3′UTR). Target sequences of SIRT4′UTR were mutated (MUT SIRT4 3′UTR). C, Dual‐luciferase reporter assay of HSC‐T6 cells transfected with WT SIRT4 3′UTR reporter together with miR‐130b‐5p mimics or NC‐miR. D, The protein levels and mRNA expression of SIRT4 were detected by Western blotting in HSC‐T6 cells transfected with antagomir or NC under the treatment of 10 ng/mL TGF‐β for 24 h. E, Western blotting analysis for the expressions of SIRT4 in liver from mice with fibrosis. F, The protein levels of SIRT4 were examined in normal liver and liver fibrosis from patients. n = 6/group. G, Immunohistochemical staining of Masson, Sirius red and SIRT4 in liver section from patients with liver fibrosis or other liver disease (n = 6/group; original magnification ×200; scale bars, 100 μm). Data represent means ± SEM of at least three independent experiments. *P < .05, **P < .01 and ***<.001. *P < .05, **P < .01 and ***P < .001

To study the molecular mechanism by which miR‐130b‐5p regulates HSC activation, the predicting software (miRbase, miRwalk and TargetScan) was used to find the target genes of miR‐130b‐5p (Figure S4). According to the results, SIRT4 may be the target of miR‐130b‐5p. Then, we examined the protein expression of SIRT4 in HSC‐T6 cells activated by TGF‐β1 for 0 hour and 24 hours. SIRT4 expression was clearly reduced in the activated HSCs (Figure 4A). A previous study showed that the overexpression of SIRT4 can prevent HFD‐induced liver fibrosis.^14^ Moreover, whether miR‐130b‐5p can regulate SIRT4 expression during HSC activation has not yet been illustrated. A luciferase reporter assay suggested that miR‐130b‐5p mimics could repress luciferase activity of SIRT4 with a wild‐type 3′‐UTR (WT SIRT4 3′‐UTR), compared with a mutant 3′‐UTR (MUT SIRT4 3′‐UTR) (Figure 4B,C). As shown in Figure 4D, knockdown of miR‐130b‐5p increased the expression of SIRT4 in HSC‐T6 cells induced with 10 ng/mL TGF‐β for 24 hours. (Figure 4D). The SIRT4 expression was reduced in the livers with fibrosis compared with controls (Figure 4E). Moreover, the protein expression levels of SIRT4 were lower in the liver fibrosis sample from patients than in those of normal controls (Figure 4F). Then, the immunochemistry result demonstrated that expression of SIRT4 was down‐regulated in the liver sample from patients with fibrosis (Figure 4G). These observations suggested that miR‐130b‐5p might target SIRT4 and regulate its expression.

### 3.5 miR‐130b‐5p regulates HSCs activation, proliferation and apoptosis via SIRT4

**FIGURE 5:**
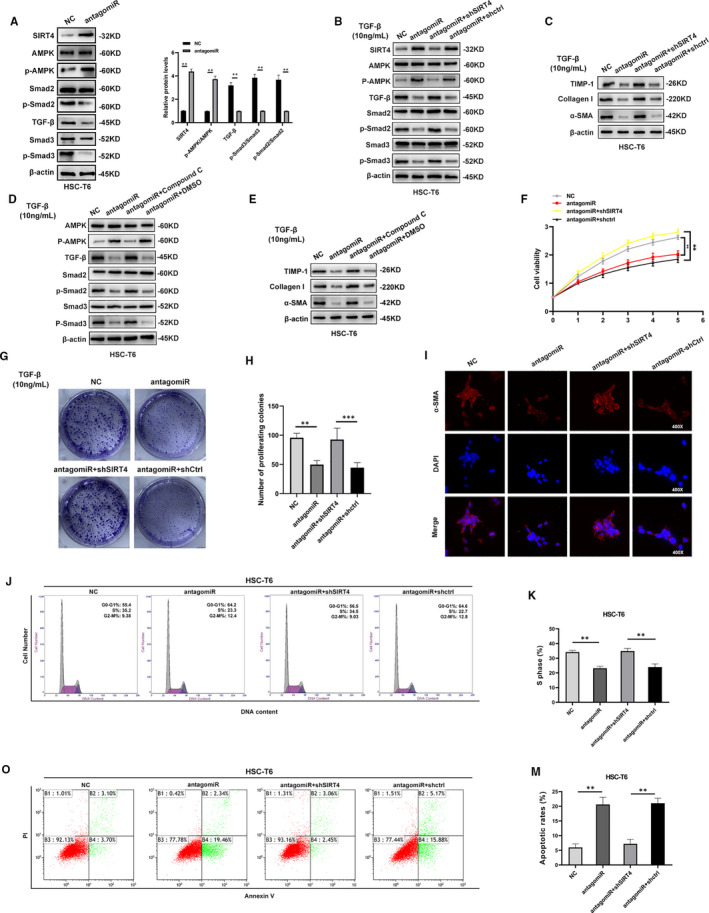
miR‐130b‐5p regulates HSC activation and proliferation via SIRT4. (A)The protein levels of SIRT4, AMPK, p‐AMPK, TGF‐β, Smad2, p‐Smad2, Smad3 and p‐smad3 were detected by Western blotting in HSC‐T6 cells induced by 10 ng/mL TGF‐β for 24 h. (B) The protein levels of SIRT4, AMPK, p‐AMPK, TGF‐β, Smad2, p‐Smad2, Smad3 and p‐Smad3 in HSC‐T6‐pre‐antagomiR cells transfected with shSIRT4 or shcontrol. (C) Western blotting analysis for the expressions of fibrotic markers in HSC‐T6‐pre‐antagomiR cells transfected with shSIRT4 or shcontrol. (D) The protein levels of SIRT4, AMPK, p‐AMPK, TGF‐β, Smad2, p‐Smad2, Smad3, p‐Smad3 in HSC‐T6‐pre‐antagomiR cells treated with compound C or DMSO. (E) Western blotting analysis for the expressions of fibrotic markers in HSC‐T6‐pre‐antagomiR cells treated with Compound C or DMSO. (F) The proliferation of HSC‐T6‐pre‐antagomiR cells transfected with shSIRT4 or shcontrol was assessed by cell viability assay, (G) colony formation assays and (H) the quantification. (I) α‐SMA (red) was identified by immunofluorescence assays in HSC‐T6‐pre‐antagomiR cells transfected with shSIRT4 or shcontrol. (J) The cell cycle distribution of HSC‐T6‐pre‐antagomiR cells transfected with shSIRT4 or shcontrol was examined by flow cytometry and (K) the quantification. (O) The cell apoptosis of HSC‐T6‐pre‐antagomiR cells transfected with shSIRT4 or shcontrol was examined by flow cytometry and (M) the quantification. Data represent means ± SEM of at least three independent experiments. *P < .05, **P < .01 and ***<.001. *P < .05, **P < .01, and ***P < .001

Previous study had reported that SIRT4 depletion augmented mTOR signalling via inactivating AMPKα.^16^ Moreover, AMPK is associated with TGF‐β/Smads pathway.^17^ Therefore, we detected the protein and mRNA expression of AMPK, p‐AMPK, TGF‐β, Smad2, p‐Smad2, Smad3 and p‐Smad3. The expression of TGF‐β, p‐Smd2 and p‐Smad3 was significantly reduced following the knockdown of miR‐130b‐5p in HSC‐T6 cells induced with 10 ng/mL TGF‐β for 24 hours, whereas SIRT4 and p‐AMPK expression was increased (Figure 5A). To further elucidate the possible mechanism by which miR‐130b‐5p modulates the activation and proliferation of HSCs by targeting SIRT4, the lentivirus was used to knockdown SIRT4 in HSC‐T6 cells transfected with antagomiR‐130b‐5p. We performed Western blot and RT‐qPCR to evaluate the efficiency of SIRT4 knockdown. The results showed that the expression level of SIRT4 in HSCs transfected with shSIRT4 is significantly lower than that of HSCs transfected with shctrl (Figure S5). TGF‐β, p‐Smad2 and p‐Smad3 expression was increased when SIRT4 expression was knocked down in HSC‐T6 cells transfected with antagomiR‐130b‐5p, but the expression levels of SIRT4 and p‐AMPK were decreased (Figure 5B). Knockdown of SIRT4 resulted in up‐regulation of pro‐fibrotic marker gene expression in HSCs transfected with antagomiR‐130b‐5p (Figure 5C). In addition, we used SIRT4 lentivirus to overexpress SIRT4 in HSC‐T6 cells transfected with antagomir‐130b‐5p and found that the expression levels of pro‐fibrotic gene were significantly lower in HSC‐T6 cells transfected with SIRT4 lentivirus compared with control. To investigate the effect of p‐AMPK on miR‐130b‐5p–mediated HSC activation, compound C was used to suppress the phosphorylation of AMPK in HSC‐T6 cells transfected with antagomiR‐130b‐5p. The data showed that phosphorylation level of AMPK was decreased. Conversely, the expression levels of TGF‐β, p‐Smad2, p‐Smad3, TIMP‐1, Collagen I and α‐SMA were significantly higher in HSCs treated with compound C than in control cells (Figure 5D,E). As shown in Figure 5E‐G, knockdown of SIRT4 promoted the cell proliferation and activation, as demonstrated by increased α‐SMA immunofluorescence under the treatment of TGF‐β (10 ng/mL) (Figure 5I). In addition, down‐regulated SIRT4 expression increased the percentage of S phase cells and decreased apoptosis in HSCs under the treatment of TGF‐β (10 ng/mL) (Figure 5J‐M and Figure S1B). Moreover, miR‐130b‐5p and SIRT4 regulated protein levels of the S phase checkpoint proteins (Figure S6). To further verify the effect of SIRT4 on liver fibrosis, HSC‐T6‐pre‐antagomiR cells were transfected with LV‐SIRT4 or LV‐NC. The results showed that overexpression of SIRT4 inhibited liver fibrosis (Figure S7). Collectively, these results suggest that miR‐130b‐5p/SIRT4 axis may exert an influence on regulating HSCs activation, proliferation and apoptosis.

### 3.6 AntagomiR‐130b‐5p ameliorates liver fibrosis in mice

**FIGURE 6:**
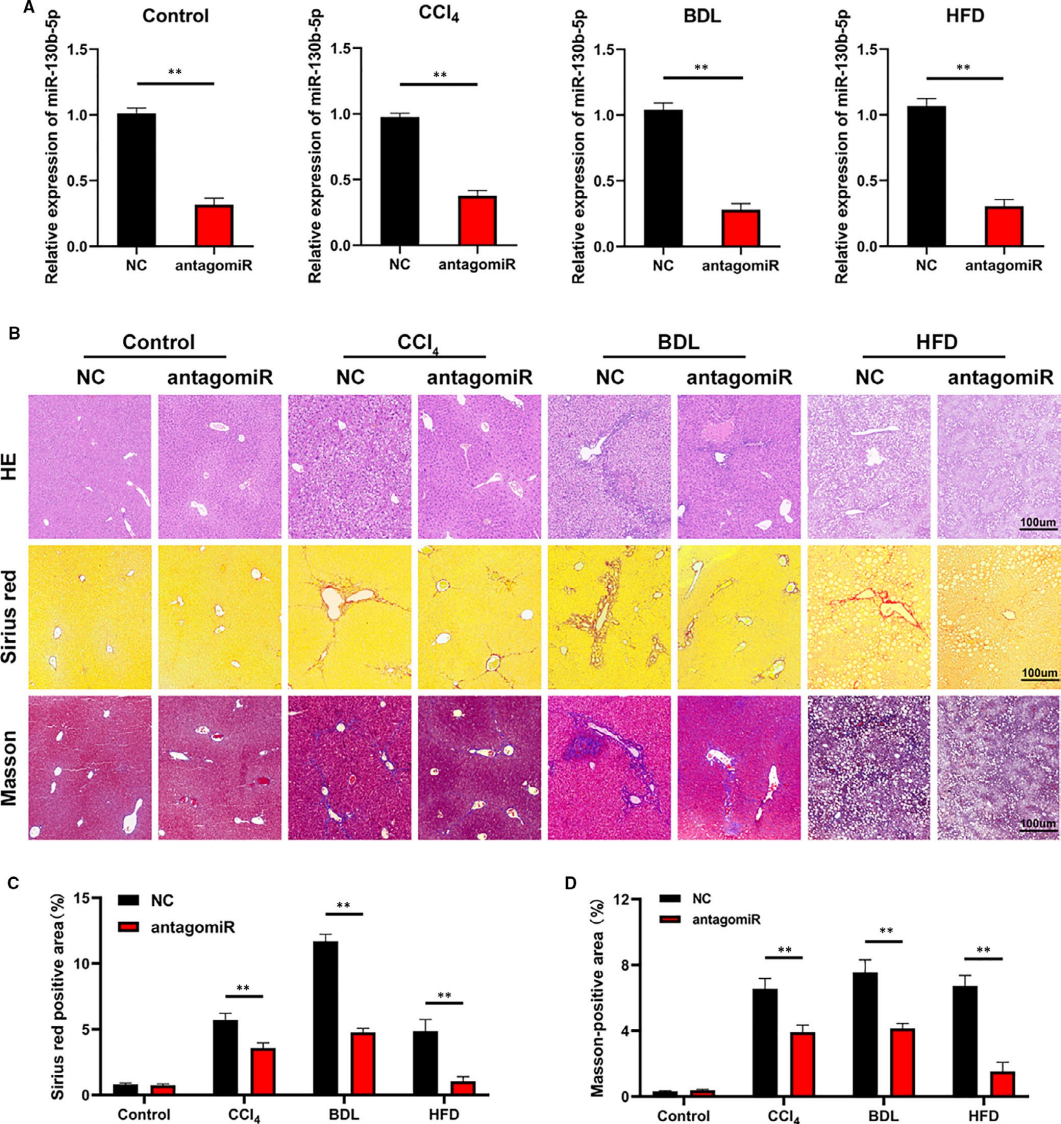
AntagomiR‐130b‐5p ameliorates liver fibrosis in mice. (A) The expression of miR‐130b‐5p was detected in mice transfected with negative control (NC) or antagomir‐130b‐5p. n = 6 mice for each group. (B) H&E, Masson and Sirius red staining of the liver section (original magnification ×20: scale bars, 100 μm). n = 6 mice for each group. (C) The quantification of Masson‐positive and (D) Sirius red‐positive fibrosis areas. n = 6 mice for each group. Graph represents mean ± SEM. *P < .05, **P < .01 and ***P < .001

Our results demonstrated that miR‐130b‐5p inhibited HSC activation and proliferation in vitro. Therefore, to verify whether antagomiR‐130b‐5p can prevent hepatic fibrosis in vivo, NC‐miR or antagomiR‐130b‐5p was injected into control mice and CCl_4_‐, BDL‐ and HFD‐treated mice. The hepatic expression of miR‐130b‐5p was measured by RT‐PCR (Figure 6A). The results of H&E, Masson and Sirius red staining suggested that the extent of liver injury and liver fibrosis was heavier in CCl_4_/BDL/HFD group mice than in control group mice (Figure 6B‐D). In general, our study indicated that antagomiR‐130b‐5p can ameliorate liver fibrosis in mice.

### 3.7 miR‐130b‐5p reduces the expression of SIRT4 in hepatic fibrosis tissues

**FIGURE 7:**
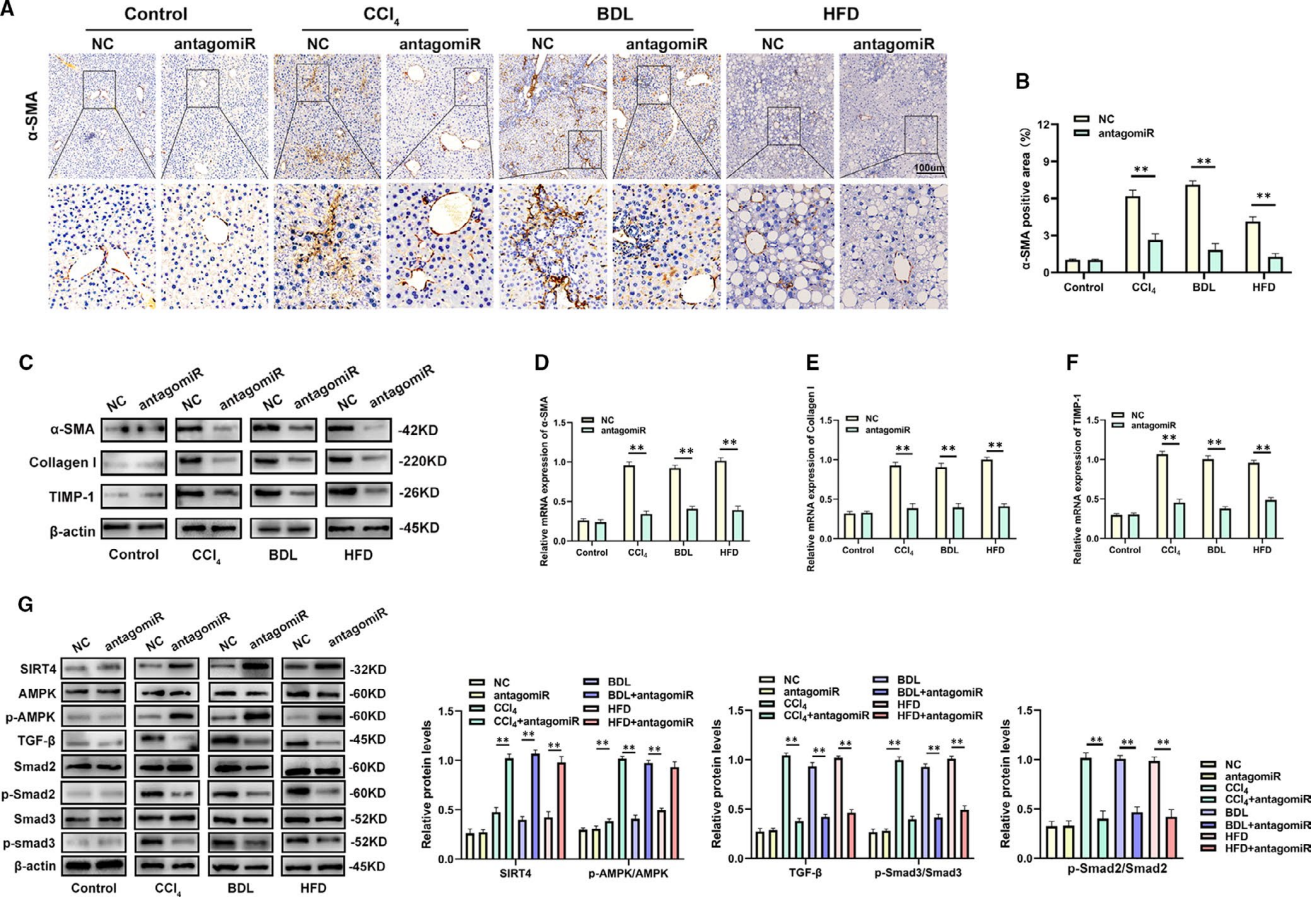
miR‐130b‐5p reduces the expression of SIRT4 in hepatic fibrosis tissues. A, α‐SMA staining of α‐SMA in liver sections from representative mice from each group. n = 6 mice for each group. B, The quantification of α‐SMA‐positive area in the livers from mice. n = 6 mice for each group. C, The fibrotic marker gene protein expression was detected in liver sections from mice transfected with NC‐miR or antagomir‐130b‐5p by Western blotting. D, The mRNA levels of α‐SMA were examined by quantitative real‐time PCR. E, The mRNA levels of Collagen I was detected by quantitative real‐time PCR. F, The mRNA levels of TIMP‐1 were detected by quantitative real‐time PCR. G, The protein levels of SIRT4, AMPK, p‐AMPK, Smad2, p‐Smad2, TGF‐β, Smad3 and p‐Smad3 in liver section was measured by western blotting. Data represent means ± SEM of at least three independent experiments. *P < .05, **P < .01 and ***<.001

Next, analysis of α‐SMA expression indicated that knockdown of miR‐130b‐5p attenuated liver fibrosis in vivo (Figure 7A,B). Consistently, in accordance with the reduction in miR‐130b‐5p levels, the expression levels of fibrotic gene were decreased (Figure 7C‐F). While the activation of Smad2, Smad3 and AMPK was inhibited, the SIRT4 protein expression was increased in the liver tissues of mice with fibrosis after treatment with antagomiR‐130b‐5p (Figure 7G). In conclusion, our findings demonstrated that miR‐130b‐5p promotes liver fibrosis by regulating SIRT4 via the AMPK/TGF‐β/Smad2/3 signalling pathway.

## 4 DISCUSSION

Hepatic fibrosis is a progressive disease accompanied by the deposition of ECM. Exposed to long‐lasting damage, liver fibrosis could result in cirrhosis, liver failure, liver cancer and ultimately death.^18^ HSCs are mainly responsible for liver fibrosis. In response to liver injury, quiescent HSCs are activated and transdifferentiate into proliferative, fibrogenic myofibroblasts, which produce most of the ECM.^19^, ^20^

Growing evidence has shown that the miRNAs are key factors in the progress of fibrosis.^21^ Aberrant miRNA levels could regulate HSC activation and liver fibrosis.^22^ Recent studies have shown that miR‐130b‐5p has different functions in diverse types of tumours.^23^, ^24^ In addition, miR‐130b‐5p was reported to ameliorate activated microglia‐induced neuronal injury via the TLR4/NF‐κB signalling pathway.^25^ A report has demonstrated that microRNA‐130b‐5p as a regulator for inhibition and treatment of NAFLD.^15^ However, the underlying mechanism of miR‐130b‐5p in HSC activation is poorly understood.

In this study, the data indicated that miR‐130b‐5p expression was significantly increased during HSC activation. Next, we observed that knockdown of miR‐130b‐5p prevented HSC activation and proliferation and promoted apoptosis of HSCs. Moreover, miR‐130b‐5p expression was up‐regulated in hepatic fibrosis in CCl_4_, BDL, and HFD models and in patients with fibrosis. Compared with controls, mice transfected with antagomiR‐130b‐5p could ameliorate liver fibrosis. Our findings in vitro and in vivo suggested that miR‐130b‐5p may play a promoting role in liver fibrogenesis and that it acts as a positive regulator in HSCs activation.

Bioinformatics analysis revealed that SIRT4 might be a direct target of miR‐130b‐5p. SIRT4, which resides mitochondria and plays a significant role in cellular metabolic processes.^26^ EX‐527, as a SIRT1‐selective inhibitor, relieve hepatic fibrosis by up‐regulating SIRT4 expression.^14^ A previous study indicated that SIRT4 silencing in tumour‐associated macrophages promotes HCC development.^27^ SIRT4 depletion augmented mTOR signalling via inactivating AMPKα.^16^ AMPK, which consists of α, β and γ subunits, is a member of a serine/threonine (Ser/Thr) kinase family and is expressed in multiple organs.^28^ The luciferase reporter assay showed that SIRT4 is a target that is bound by miR‐130b‐5p. Former studies have verified that overexpression of SIRT4 can activate AMPKα.^16^ As expected, we found that the expression levels of p‐AMPK were decreased in HSCs transfected with antagomiR‐130b‐5p. The previous report have proved that AMPK protects against fibrosis in the heart,^9^ liver,^29^ lung,^30^ kidney^31^ and skin.^32^ Activation of AMPK can repress fibroblast proliferation and extracellular matrix accumulation and inhibit the transforming growth factor‐β (TGF‐β)/Smads signalling pathway.^17^ TGF‐β is an important cytokine that performs a key role in regulating tissue development and homeostasis.^33^ Moreover, the TGF‐β signalling pathway is closely related to the activation of HSCs^34^ and induces ECM synthesis,^35^ and its effector Smad proteins (Smad2, Smad3, Smad4) exert various and even opposing functions in the regulation of fibrosis.^35^ Smad2, an antifibrotic role, is a major regulator upon the pro‐fibrotic function of Smad3.^35^ Smad4, as a pro‐fibrotic protein, can promote collagen I promoter activity.^36^ Our study indicated that knockdown of miR‐130b‐5p can alleviate hepatic fibrosis by targeting SIRT4 via the AMPK/TGF‐β/Smad2/3 signalling pathway.

**FIGURE 8:**
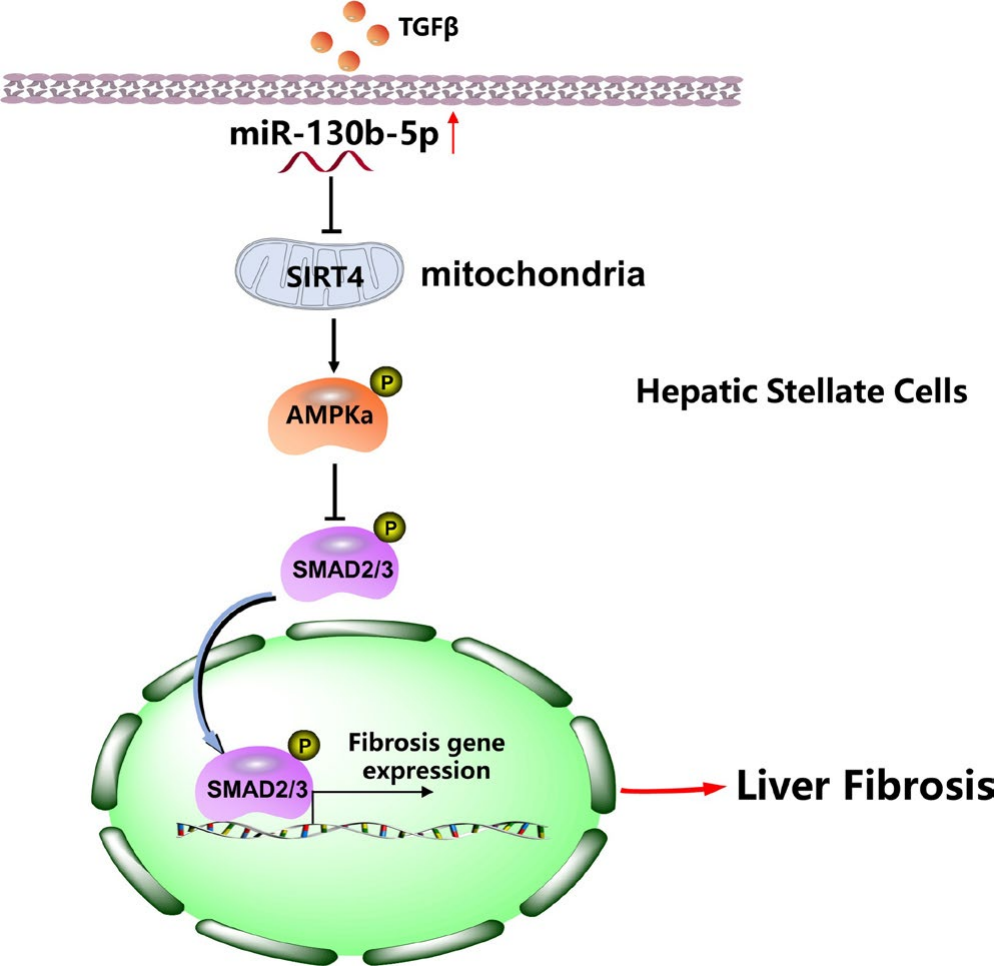
Schematic diagram showing the mechanism of miR‐130b‐5p–mediated liver fibrosis. miR‐130b‐5p could be up‐regulated in liver fibrosis models, miR‐130b‐5p overexpression inhibited SIRT4 expression and phosphorylation of AMPK, and it resulted in promoting phosphorylation of Smad2 and Smad3 to accelerate the progression of liver fibrosis

Collectively, our study revealed that the expression of miR‐130b‐5p was significantly up‐regulated in activated HSCs and liver fibrosis models. miR‐130b‐5p, as a pro‐fibrotic factor, promoted the expression of pro‐fibrotic markers and HSC activation by targeting SIRT4 via the AMPK/TGF‐β/Smad2/3 signalling pathway (Figure 8). Furthermore, antagomiR‐130b‐5p ameliorated liver fibrosis and promoted SIRT4 expression in animal models. Indeed, lack of substantial molecular mechanism, how SIRT4 activate AMPK, is one of the limitations of our study. We would address this question in our future research. In general, our study revealed a mechanism by which miR‐130b‐5p is involved in the activation of HSCs, and it offers a possible miRNA‐based therapeutic strategy for alleviating hepatic fibrosis.

## DATA AVAILABILITY STATEMENT

The raw data supporting the conclusions of this article will be made available by the authors, without undue reservation.

## CONFLICT OF INTEREST

The authors declare there is no conflict of interest.

## AUTHOR CONTRIBUTIONS

**Hao Wang:** Conceptualization (equal); Data curation (equal); Writing‐original draft (equal). **Zeng Wang:** Data curation (equal); Resources (equal); Software (equal). **Yirui Wang:** Supervision (equal); Validation (equal). **Xiangcheng Li:** Conceptualization (equal); Writing‐review & editing (equal). **Wenjie Yang:** Supervision (equal); Validation (equal). **Song Wei:** Conceptualization (equal); Writing‐review & editing (equal). **Chengyu Shi:** Visualization (equal). **Jiannan Qiu:** Validation (equal). **Ming Ni:** Conceptualization (equal); Writing‐review & editing (equal). **Jianhua Rao:** Conceptualization (equal); Writing‐review & editing (equal). **Feng Cheng:** Conceptualization (equal); Funding acquisition (equal).

## Supporting information

Fig S1‐S7

Tab S1
