# Supplementary material for: miRNA‐130b‐5p promotes hepatic stellate cell activation and the development of liver fibrosis by suppressing SIRT4 expression
Source: J Cell Mol Med. 2021 Jul 17;25(15):7381–94. doi: 10.1111/jcmm.16766 (PMC8335697; doi:10.1111/jcmm.16766)
Supplement: Supplementary file 1 — Fig S1‐S7 [file JCMM-25-7381-s001.docx]

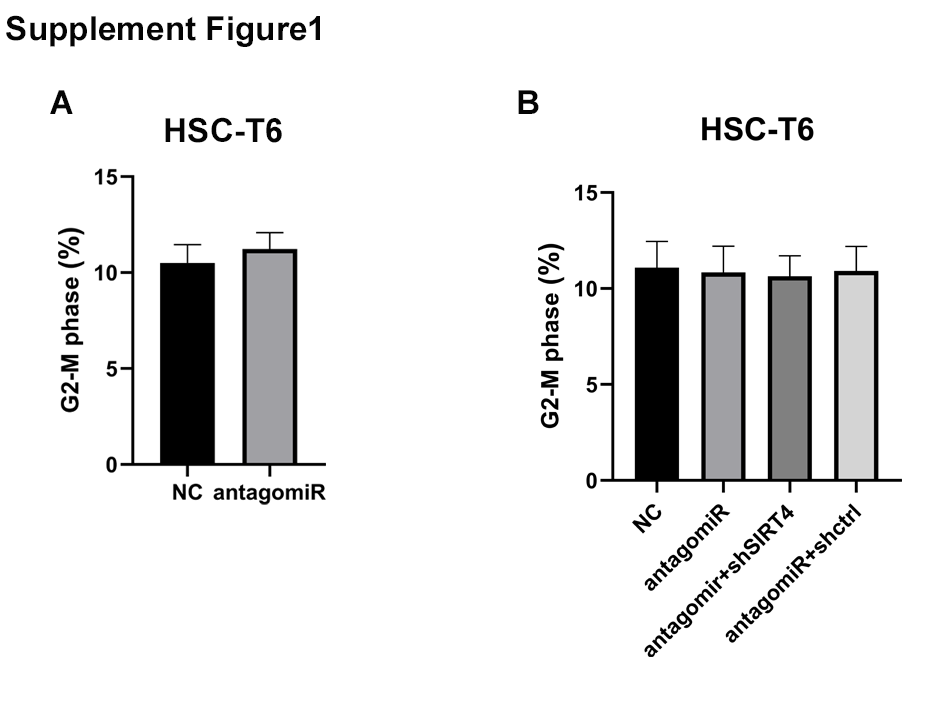


**Supplement Figure 1. The upregulation of population of G2/M phase (from about 9% to 12%) is not statistically significant increase.**

**(A)** The population of G2/M phase from three experiment was calculated in Fig 3G. **(B)** The population of G2/M phase from three experiment was calculated in Fig 5J**.** Data represent means±SEM of at least three independent experiments. *p < 0.05 and **p < 0.01.


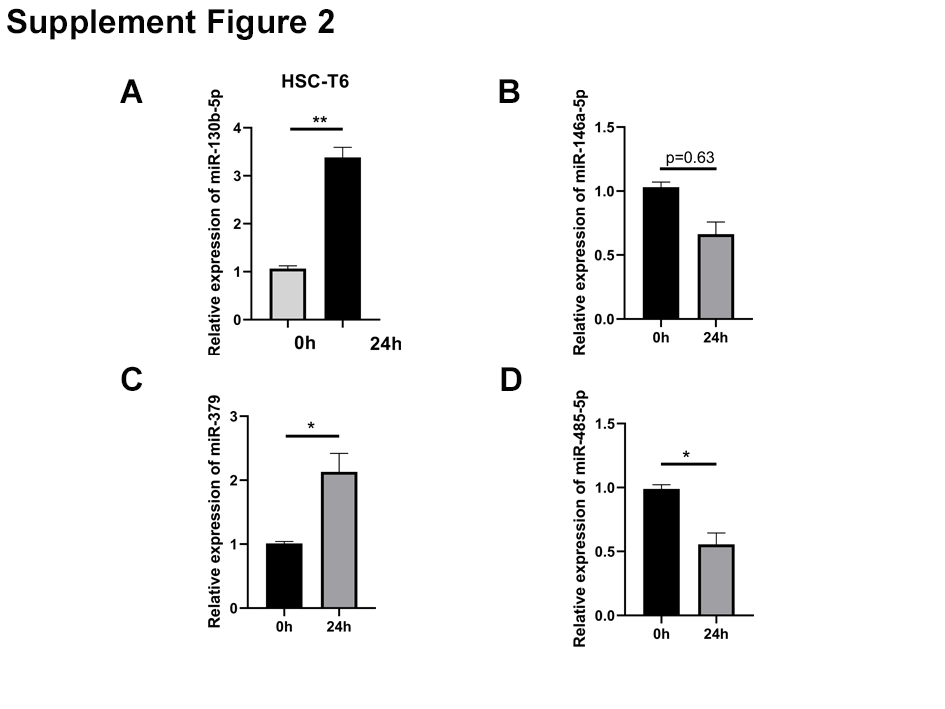


**Supplement Figure 2. The expression level of miR-130b-5p is most significantly upregulated in the miRNAs.**

**(A)-(D)** The miRNA expression levels were measured by qRT-PCR**.** Data represent means±SEM of at least three independent experiments. *p < 0.05 and **p < 0.01.


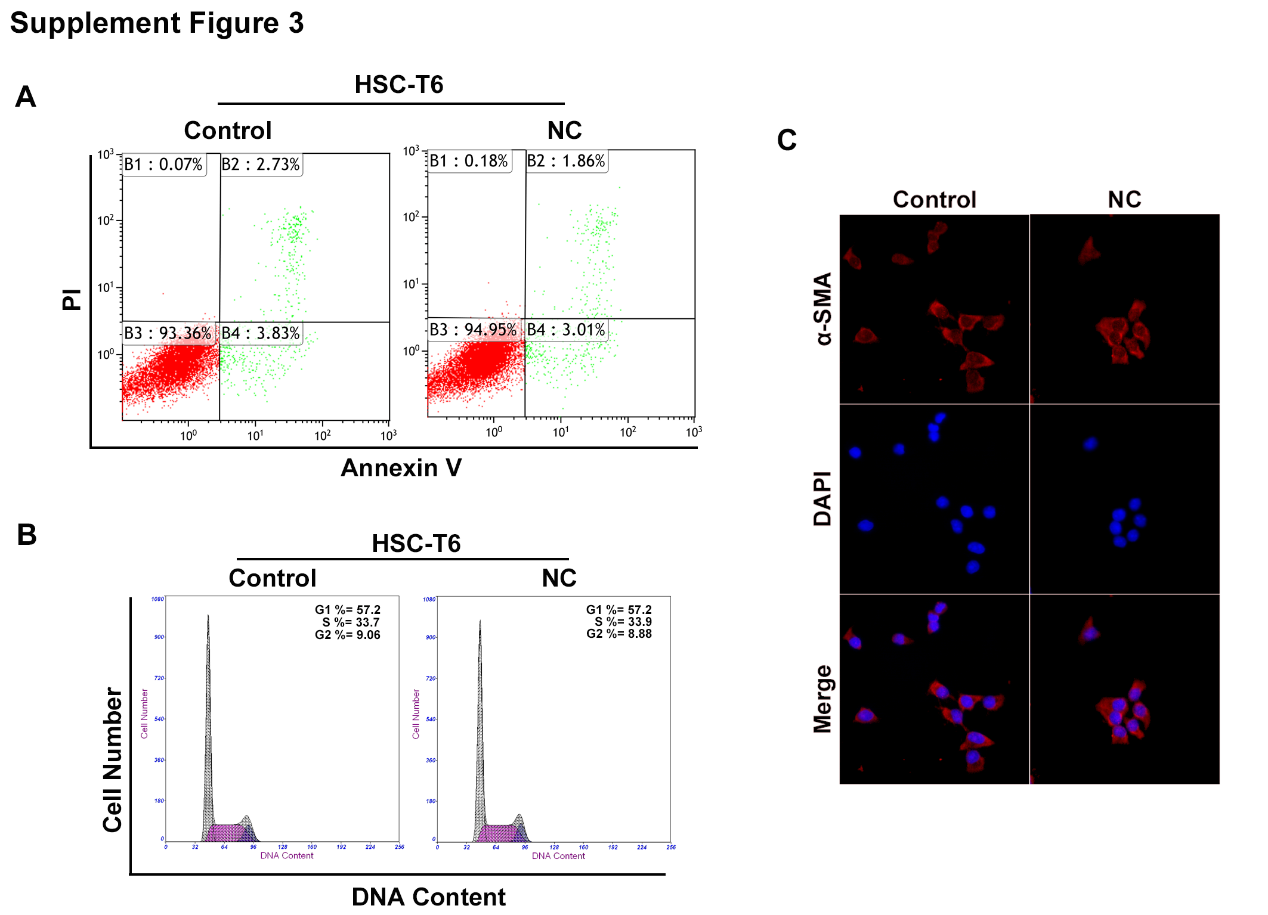


**Supplement Figure 3. The results of cell proliferation, cell apoptosis and immunofluorescence in untransfected cells or transfected cells.**

**(A)** The cell apoptosis of cells transfected with NC-miR or cells untransfected was measured by flow cytometry. **(B)** The cell-cycle distribution of cells transfected with NC-miR or cells untransfected measured by flow cytometry. **(C)** α-SMA (red) was identified by immunofluorescence assays in HSC-T6 cells transfected NC-miR or HSC-T6 cells untransfected. Data represent means±SEM of at least three independent experiments. *p < 0.05 and **p < 0.01.


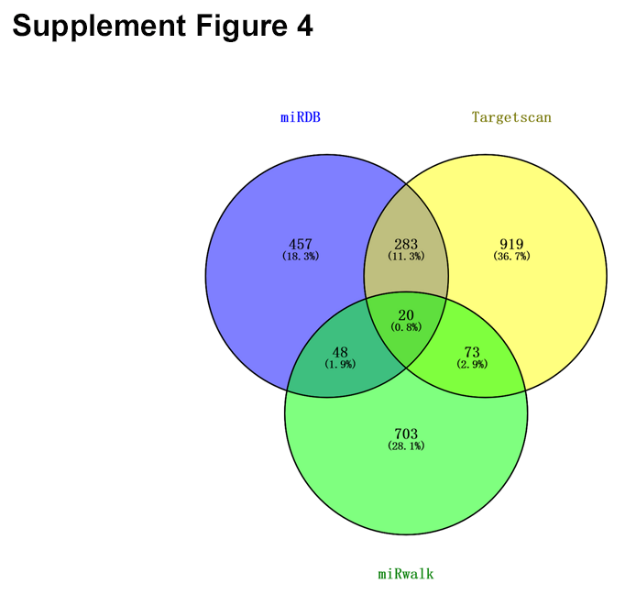


**Supplement Figure 4. The target genes of miR-130b-5p were predicted by software.**

The venn diagram showed the overlapped target genes.


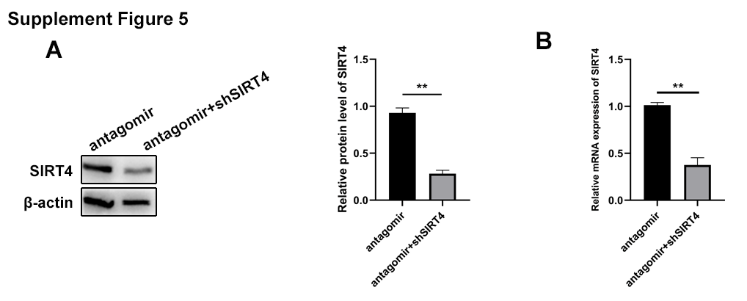


**Supplement Figure 5. SIRT4 was silenced by shSIRT4.**

**(A)** The protein expression of SIRT4 was detected by western blot. **(B)** The mRNA level of SIRT4 was measured by qRT-PCR. Data represent means±SEM of at least three independent experiments. *p < 0.05 and **p < 0.01.


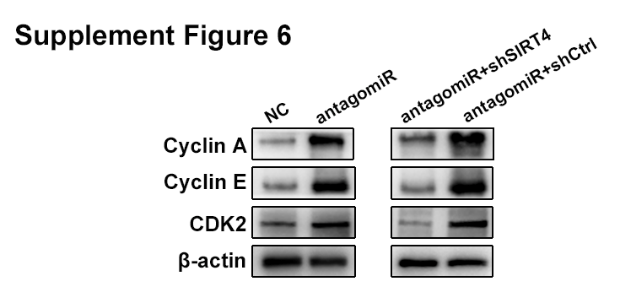


**Supplement Figure 6. miR-130b-5p and SIRT4 regulated protein levels of the S phase checkpoint proteins.**

The protein levels of CDK2, Cyclin A and Cyclin E was detected by western blot. Data represent means±SEM of at least three independent experiments.


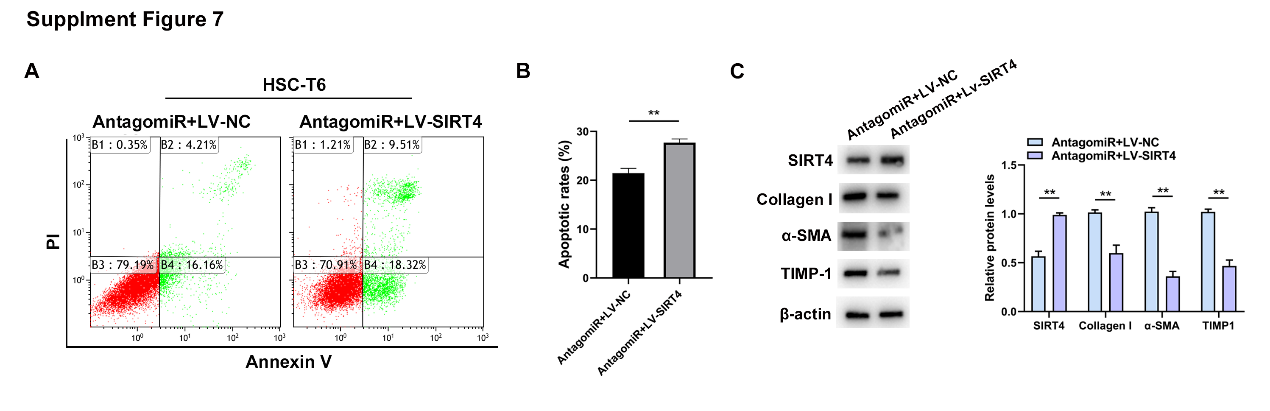


**Supplement Figure 7. Overexpression of SIRT4 inhibited liver fibrosis.**

**(A)** The cell apoptosis of HSC-T6-pre-antagomiR cells transfected with LV-SIRT4 or NC was measured by flow cytometry and **(B)** the quantification. **(C)** The protein expression of SIRT4 was detected by western blot. Data represent means±SEM of at least three independent experiments.
