# Supplementary material for: miRNA‐130b‐5p promotes hepatic stellate cell activation and the development of liver fibrosis by suppressing SIRT4 expression
Source: J Cell Mol Med. 2021 Jul 17;25(15):7381–94. doi: 10.1111/jcmm.16766 (PMC8335697; doi:10.1111/jcmm.16766)
Supplement: Supplementary file 2 — Tab S1 [file JCMM-25-7381-s002.docx]

Supplementary table 1: Primer sequences for the amplification.

| Gene | Forward Primer（5′ → 3′） | Reverse Primer（5′ → 3′） |
| --- | --- | --- |
| SIRT4(Mouse) | CAGATGTCGTTTTCTTCG | CCAGAGTATACCTGCAAGG |
| miR-130b-5p (Mouse) | GCGCCTCATCACGTTGTCCCTTTCTCA | GTGCAGGGTCCGAGGT |
| U6 (Mouse) | GCTTCGGCACATATACTAAAAT | CGCTTCACGAATTTGCGTGTCAT |
| α-SMA (Mouse) | GTCCCAGACATCAGGGAGTAA | TCGGATACTTCAGCGTCAGGA |
| Collagen-I (Mouse) | GCTCCTCTTAGGGGCCACT | CCACGTCTCACCATTGGGG |
| TIMP-1 (Mouse) | GCAACTCGGACCTGGTCATAA | CGGCCCGTGATGAGAAACT |
| β-actin (Mouse) | GGCTGTATTCCCCTCCATCG | CCAGTTGGTAACAATGCCATGT |
